# Poorly differentiated cluster grade-a vital predictor for lymph node metastasis and oncological outcomes in patients with T1 colorectal cancer: a retrospective study

**DOI:** 10.1186/s12876-022-02492-7

**Published:** 2022-09-05

**Authors:** Xiaolin Ji, Mei Kang, Xianzhi Zhao, Xiaoyu Li, Yingjie Guo, Ping Xie, Yanan Yu, Zibin Tian

**Affiliations:** 1 Department of Gastroenterology, Affiliated Hospital of Qingdao University, No. 16 Jiangsu Road, Qingdao, 266003 Shandong China; 2 Department of Internal Medicine, Weicheng District Weifang City Peoples Hospital, Weifang, Shandong China

**Keywords:** Poorly differentiated cluster grade, Predictor, T1 colorectal cancer, Lymph node metastasis, Oncological outcomes

## Abstract

**Background:**

T1 colorectal cancers have a low lymph node metastasis rate and good prognosis. Thus, endoscopic resection is an attractive choice. This study aimed to describe the value of poorly differentiated cluster grade in identifying endoscopically curable T1 colorectal cancers.

**Methods:**

We included 183 T1 colorectal cancer patients who underwent curative resection. Univariate and multivariate logistic regressions were used to identify lymph node metastasis predictors. The Akaike information criterion was used to determine whether poorly differentiated cluster grade was the best predictor. Backward regression was used to screen the variables. Survival analyses were conducted to determine the prognostic predictive power of poorly differentiated cluster grade. Correlations among predictors and concordance between our pathologists were also investigated.

**Results:**

Poorly differentiated cluster grade was an independent predictor for lymph node metastasis (adjusted odds ratio [OR]_G 3_ = 0.001; 95% confidence interval [95% CI]_G 3_ =  < 0.001, 0.139) in T1 colorectal cancer patients; moreover, it had the best predictive value (AIC = 61.626) among all indicators. It was also screened for inclusion in the predictive model. Accordingly, a high poorly differentiated cluster grade independently indicated shorter overall survival (hazard ratio [HR]_G 2_ = 4.315; 95% CI_G 2_ = 1.506, 12.568; HR_G 3_ = 5.049; 95% CI_G 3_ = 1.326, 19.222) and disease-free survival (HR_G 3_ = 6.621; 95% CI_G 3_ = 1.472, 29.786).

**Conclusions:**

Poorly differentiated cluster grade is a vital reference to manage T1 colorectal cancer. It could serve as an indicator to screen endoscopically curable T1 colorectal cancers.

## Background

Colorectal cancer (CRC) is the third most common cancer and the second leading cause of cancer-related deaths worldwide [1, 2], and its mortality rate has increased year over year in young adults [3]. Screening programs have increased the diagnostic rate of early colorectal cancer (ECRC) [4, 5]; fortunately, the 5-year survival rate for patients who are diagnosed with localized CRC is nearly 90% [6]. This suggests that some cancer-specific deaths might be preventable. Epidemiological data have confirmed assumptions that CRC mortality has declined as screening rates have increased [7]. However, the dilemma of choosing a suitable treatment strategy then arises. A low lymph node metastasis (LNM) rate (ranging from 0 to 17% [8–10]) is one of the characteristics of T1 CRCs; however, direct removal via surgery remains controversial, and more conservative methods, such as endoscopic submucosal dissection (ESD), might be appropriate substitutions [11–13]. Conservative therapeutic approaches can lead to undertreatment and insufficient evaluations of tumor stage, even though they can reduce perioperative and postoperative complications [14] and improve patient quality of life. In addition, approximately 10–25% of patients with early-stage CRC and those who do not receive adjuvant therapy were found to progress unexpectedly during follow-up [15, 16]. Consequently, accurately predicting LNM in T1 CRC patients to determine whether the tumor can be curatively resected under endoscopy [17] could guide clinicians in appropriate patient management. Investigators have already established risk stratification models based on tumor morphology, lesion size, and the other parameters, but these models are unsatisfactory, with nearly 90% of patients classified as high-risk having undergone unnecessary surgeries [18, 19]. Therefore, a more accurate indicator/model is urgently needed. To this end, poorly differentiated clusters (PDCs) have become our research focus. PDCs are clusters/nests of 5 or more tumor cells that can be found in the invasive front and stroma of tumors [20]. Pathologists have further subdivided them into grades, G1 (0–4 clusters), G2 (5–9 clusters), and G3 (≥ 10 clusters), which can be determined under an objective lens with a magnification of × 20 [20–22]. The predictive value of PDCs and PDC grade has been demonstrated not only in CRC patients [21, 23, 24] but also in patients with other diseases [25]. The presence of PDCs or a high PDC grade is indicative of an unfavorable prognosis [10, 20, 21, 24, 26–28]. Additionally, excellent interobserver agreement has been achieved for PDCs and PDC grade, which has improved its clinical application [20, 27–29]. However, the results of a large number of previous studies are not suitable to reference for the management of T1 CRC patients because several of these works are based on stage II and III CRC patients [27, 30]; investigations into T1 CRC patients have rarely mentioned their oncological outcomes [10].

We aimed to clarify the value of the PDC grade in determining T1 CRCs requiring surgery. When the risk of LNM is considered minimal in patients with T1 CRC, endoscopic resection is an effective alternative to radical resection. We investigated the influence of PDC grade on LNM and compared its predictive power with that of other indicators. Moreover, we developed and tested a proper model based on the PDC grade and illustrated its unique role in clinical settings. Oncological outcomes among T1 CRC patients were examined to validate the prognostic value of the PDC grade. We also identified and described some correlations among selected parameters that are worthy of mention. Additionally, finding the interobserver agreement for our PDC grading was one of our objectives.

## Methods

### Ethics approval

This research study was conducted retrospectively from data obtained for clinical purposes and was reviewed and approved by the Ethics Committee of the Affiliate Hospital of Qingdao University (Reference Number QYFYWZLL26957). The procedures used in this study adhere to the tenets of the Declaration of Helsinki. The need for written informed consent was waived by the Ethics Committee of the Affiliate Hospital of Qingdao University due to retrospective nature of the study.

### Patients and parameters

The data from patients who underwent curative operations for primary T1 CRC at the Affiliated Hospital of Qingdao University from 2002 to 2020 were retrospectively reviewed and analyzed. All resections were primary surgical resections. Our mean number of lymph nodes (LNs) per patient was 15, when the American Joint Committee on Cancer Staging (AJCC) and National Quality Forum guidelines suggested that at least 12 LNs should be dissected for CRC patients [31]. Moreover, the dissections of para-colorectal LNs and regional nodes along the named vessels were given to all patients. All LN dissection performed to the center involved central vessel ligation (CVL), and all rectal lesions underwent total mesorectal excision (TME). The distances from the surgical margins to the edge of the tumor were at least 10 cm in both the proximal and distal ends for colon cancers and at least 5 cm and 2 cm to the upper and lower edges of rectal cancers, respectively. The exclusion criteria were synchronous multiple cancers (n = 8), preoperative distant metastasis (n = 5), treatment with neoadjuvant chemoradiotherapy (n = 69), and missing data (n = 6). Ultimately, 183 patients were included.

Potential variables included the PDC grade, depth of submucosal invasion (> 1 mm vs. ≤ 1 mm), stratification of submucosal invasion (SM 1 vs. SM 2), lesion size (> 20 mm vs. ≤ 20 mm), tumor volume (the mean volume was the cutoff), tumor budding (TB) grade, World Health Organization tumor grade (for the entire tumor, abbreviated as tumor grade in this work), perineural invasion (PNI), lymphvascular invasion (LVI), mucinous composition, tumor location (right colon vs. left colon vs. rectum), number of lesions (unifocal vs. multifocal), operation method (laparotomy vs. laparoscopy), and postoperative adjuvant chemotherapy (for patients survival). Patient age, sex, body mass index (BMI), hypertension (HP), diabetes mellitus (DM), and coronary heart disease (CHD) are also shown.

### Pathological evaluation

All tumor tissues were independently reassessed by at least two experienced pathologists who were blinded to the clinical features and original histological reports. In cases of disagreement, a third pathologist (or more) joined the assessment. Majority decisions were considered the final consensus. Moreover, our interobserver concordance of PDC grading was also tested. Pathological parameters were determined by gross specimen analysis rather than endoscopic biopsy to prevent biases caused by insufficient samples. The number of PDCs in a single field with the highest activity was determined and subsequently graded. TB was defined as 1–4 malignant cells at the tumor invasive front, which were evaluated in one field using a 20 × objective lens and counted according to the International Consortium of TB Recommendations (ITBCC) [32, 33]. TB was graded as Bd1 (0–4 buds), Bd2 (5–9 buds) or Bd3 (≥ 10 buds). To ensure the deepest portion of the invasive front was included, all available hematoxylin and eosin (H&E)-staining slides that included full-thickness sections of the tumor (mean, 5 tumor slides/patient; range, 1 to 13 slides/patient) were reviewed. Other pathological markers were also reinvestigated, namely, tumor grade, LVI, and PNI.

### Outcomes

We used a composite endpoint. The primary outcome was the implication of the parameters (especially PDC grade) on LNM status. The secondary endpoint was the prognostic value of the identified indexes, including overall survival (OS) and disease-free survival (DFS). OS was defined as the date of surgery to the date of death or the follow-up deadline (April 30, 2021). DFS was defined as the date of surgery to the date of recurrence/distant-metastasis or the follow-up deadline (April 30, 2021).

### Follow-up

Postoperative outcomes were investigated through routine scheduled outpatient visits at 3-month intervals during the first 2 years, at 6-month intervals for 3–5 years, and at 12-month intervals thereafter. The following examinations were performed: colonoscopy, abdominal and pelvic computed tomography, and measurement of serum tumor markers levels. In addition, the investigators conducted telephone interviews to estimate the general condition of each patient (recurrence vs. distant-metastasis vs. mortality).

### Statistical analysis

Univariate and multivariate logistic regressions were used to analyze the primary endpoint. Variables that were significant in univariate analyses were introduced into the multivariate analysis. The LNM fit was compared by the Akaike information criterion (AIC) value (the smaller the value is, the better the fit). Backward regression was used to explore the models. Survival curves were plotted by Kaplan–Meier survival curves (K-M curves), and the differences were evaluated with a log-rank test. Univariate analyses selected covariates to introduce into the multivariate Cox regression models to identify independent risk factors for a poor prognosis. Correlations among variables were evaluated by the χ^2^ test or Fisher’s exact test. Moreover, our interobserver concordance of the PDC grading system was shown by the Kappa consistency test to validate the effectiveness of PDC as a predictor. All statistical analyses were conducted with SPSS software (version 25.0, SPSS). A *P value* < 0.05 (two-sided) was considered statistically significant.

## Results

### Baseline characteristics of the patients with T1 CRCs

A cohort of 183 patients was included and analyzed in this study. The LNM rate was 10.9%. The percentages of patients with different PDC grades were G1, 71.6% (131/183); G2, 19.7% (36/183); and G3, 8.7% (16/183). Among the patients with LNM, the percentages of patients with different PDC grades were G1, 5.0% (1/20); G2, 30.0% (6/20); and G3, 65.0% (13/20). Among the patients without LNM, the percentages of patients with different PDC grades were G1, 79.8% (130/163); G2, 18.4% (30/163); and G3, 1.8% (3/163).

Patients with LNM tended to have a higher PDC grade (*P* < 0.001), deeper submucosal invasion (*P* < 0.001), higher TB grade (*P* < 0.001), PNI (*P* < 0.001), LVI (*P* = 0.002), and postoperative adjuvant chemotherapy (*P* < 0.001) (Table 1). The factors found more often in high PDC grade patients were deeper submucosal invasion (*P* < 0.001), deeper submucosal stratification (*P* = 0.008), higher TB grade (*P* < 0.001), PNI (*P* = 0.003), LVI (*P* = 0.001), greater mucinous composition (*P* = 0.015), and postoperative adjuvant chemotherapy (*P* < 0.001) (see details in Table 2).

**Table 1:** Clinicopathological parameters correlated with lymph node metastasis

Parameters	LNM		*P*
	Positive (%)	Negative (%)	
PDC grade			< 0.001
G 1	1 (5.0)	130 (79.8)	
G 2	6 (30.0)	30 (18.4)	
G 3	13 (65.0)	3 (1.8)	
Depth of submucosal invasion			< 0.001
> 1 mm	17 (85.0)	43 (26.4)	
≤ 1 mm	3 (15.0)	120 (73.6)	
Stratification of submucosal invasion			0.535
SM 1	2 (10.0)	30 (18.4)	
SM 2	18 (90.0)	133 (81.6)	
Tumor size (mm)			0.565
> 20	5 (25.0)	51 (31.3)	
≤ 20	15 (75.0)	112 (68.7)	
Tumor volume (mm^3^)			0.432
> 583.96	3 (15.0)	37 (22.7)	
≤ 583.96	17 (85.0)	126 (77.3)	
TB grade			< 0.001
Bd 1	3 (15.0)	128 (78.5)	
Bd 2	8 (40.0)	30 (18.4)	
Bd 3	9 (45.0)	5 (3.1)	
Tumor grade (WHO)			0.364
1	2 (10.0)	30 (18.4)	
2	17 (85.0)	111 (68.1)	
3	1 (5.0)	22 (13.5)	
PNI			< 0.001
Absence	13 (65.0)	154 (94.5)	
Presence	7 (35.0)	9 (5.5)	
LVI			0.002
Absence	14 (70.0)	154 (94.5)	
Presence	6 (30.0)	9 (5.5)	
Mucinous composition			0.463
Absence	3 (15.0)	17 (10.4)	
Presence	17 (85.0)	146 (89.6)	
Location			0.386
Right colon	0 (0.0)	15 (9.2)	
Left colon	5 (25.0)	32 (19.6)	
Rectum	15 (75.0)	116 (71.2)	
Ulceration			0.516
Absence	16 (80.0)	139 (85.3)	
Presence	4 (20.0)	24 (14.7)	
Number of lesions			0.443
Unifocal	19 (95.0)	159 (97.5)	
Multifocal	1 (5.0)	4 (2.5)	
Operation method			0.540
Laparotomy	8 (40.0)	77 (47.2)	
Laparoscopy	12 (60.0)	86 (52.8)	
Postoperative adjuvant chemotherapy			< 0.001
Absence	1 (5.0)	160 (98.2)	
Presence	19 (95.0)	3 (1.8)	
Age			0.229
≤ 60	10 (50.0)	104 (63.8)	
> 60	10 (50.0)	59 (36.2)	
Sex			0.276
Male	10 (50.0)	61 (37.4)	
Female	10 (50.0)	102 (62.6)	
BMI			0.249
≤ 28	18 (90.0)	129 (79.1)	
> 28	2 (10.0)	34 (20.9)	
HP			0.657
Absence	12 (60.0)	106 (65.0)	
Presence	8 (40.0)	57 (35.0)	
DM			0.211
Absence	19 (95.0)	138 (84.7)	
Presence	1 (5.0)	25 (15.3)	
CHD			0.294
Absence	17 (85.0)	150 (92.0)	
Presence	3 (15.0)	13 (8.0)	

*LNM* lymph node metastasis, *PDC* poorly differentiated cluster, *TB* tumor budding, *PNI* perineural invasion, *LVI* lymphvascular invasion, BMI body mass index, *HP* hypertension, *DM* diabetes mellitus, *CHD* coronary heart disease

**Table 2:** Clinicopathological parameters correlated with poorly differentiated cluster grade

Parameters	PDC grade			*P*
	G 1 (%)	G 2 (%)	G 3 (%)	
Depth of submucosal invasion				< 0.001
> 1 mm	25 (19.1)	20 (55.6)	15 (93.8)	
≤ 1 mm	106 (80.9)	16 (44.4)	1 (6.3)	
Stratification of submucosal invasion				0.008
SM 1	30(22.9)	2 (5.6)	0 (0.0)	
SM 2	101 (77.1)	34 (94.4)	16 (100.0)	
Tumor size (mm)				0.470
> 20	37 (28.2)	14 (38.9)	5 (31.3)	
≤ 20	94 (71.8)	22 (61.1)	11 (68.8)	
Tumor volume (mm^3^)				0.596
> 583.96	29 (22.1)	9 (25.0)	2 (12.5)	
≤ 583.96	102 (77.9)	27 (75.0)	14 (87.5)	
TB grade				< 0.001
Bd 1	114 (87.0)	16 (44.4)	1 (6.3)	
Bd 2	15 (11.5)	15 (41.7)	8 (50.0)	
Bd 3	2 (1.5)	5 (13.9)	7 (43.8)	
Tumor grade (WHO)				0.457
1	25 (19.1)	5 (13.9)	2 (12.5)	
2	89 (67.9)	25 (69.4)	14 (87.5)	
3	17 (13.0)	6 (16.7)	0 (0.0)	
PNI				0.003
Absence	125 (95.4)	30 (83.3)	12 (75.0)	
Presence	6 (4.6)	6 (16.7)	4 (25.0)	
LVI				0.001
Absence	126 (96.2)	30 (83.3)	12 (75.0)	
Presence	5 (3.8)	6 (16.7)	4 (25.0)	
Mucinous composition				0.015
Absence	9 (6.9)	8 (22.2)	3 (18.8)	
Presence	122 (93.1)	28 (77.8)	13 (81.3)	
Location				0.149
Right colon	14 (10.7)	1 (2.8)	0 (0.0)	
Left colon	26 (19.8)	5 (13.9)	6 (37.5)	
Rectum	91 (69.5)	30 (83.3)	10 (62.5)	
Ulceration				0.101
Absence	115 (87.8)	7 (19.4)	5 (31.3)	
Presence	16 (12.2)	29 (80.6)	11 (68.8)	
Number of lesions				0.413
Unifocal	128 (97.7)	35 (97.2)	15 (93.8)	
Multifocal	3 (2.3)	1 (2.8)	1 (6.3)	
Operation method				0.402
Laparotomy	59 (45.0)	20 (55.6)	6 (37.5)	
Laparoscopy	72 (55.0)	16 (44.4)	10 (62.5)	
Postoperative adjuvant chemotherapy				< 0.001
Absence	130 (99.2)	29 (80.6)	2 (12.5)	
Presence	1 (0.8)	7 (19.4)	14 (87.5)	
Age				0.568
≤ 60	48 (36.6)	13 (36.1)	8 (50.0)	
> 60	83 (63.4)	23 (63.9)	8 (50.0)	
Sex				0.052
Male	87 (66.4)	16 (44.4)	9 (56.3)	
Female	44 (33.6)	20 (55.6)	7 (43.8)	
BMI				0.097
≤ 28	107 (81.7)	25 (69.4)	15 (93.8)	
> 28	24 (18.3)	11 (30.6)	1 (6.3)	
HP				0.671
Absence	86 (65.6)	21 (58.3)	11 (68.8)	
Presence	45 (34.4)	15 (41.7)	5 (31.3)	
DM				0.802
Absence	111 (84.7)	32 (88.9)	14 (87.5)	
Presence	20 (15.3)	4 (11.1)	2 (12.5)	
CHD				0.119
Absence	123 (93.9)	30 (83.3)	14 (87.5)	
Presence	8 (6.1)	6 (16.7)	2 (12.5)	

*PDC* poorly differentiated cluster, *TB* tumor budding, *PNI* perineural invasion, *LVI* lymphvascular invasion, *BMI* body mass index, *HP* hypertension, *DM* diabetes mellitus, *CHD* coronary heart disease

### LNM implications for PDC grade

PDC grade can independently influence LNM in T1 CRC patients. Univariate logistic regressions found that PDC grade was an influencing factor for LNM (*P*_Total_ < 0.001; crude odds ratio [crude OR]_G 2_ = 0.038; 95% confidence interval [95% CI]_G 2_ = 0.004, 0.331; *P*_G 2_ = 0.003; crude OR_G 3_ = 0.002; 95% CI_G 3_ =  < 0.001, 0.018; *P*_G 3_ < 0.001) (Table 3). Multivariate analysis further revealed that PDC grade was an independent predictor for LNM (*P*_Total_ = 0.004; adjusted odds ratio [adjusted OR]_G 3_ = 0.001; 95% CI_G 3_ =  < 0.001, 0.139; *P*_G 3_ = 0.007) (Table 4).

**Table 3:** Univariate analyses of the potential predictors of lymph node metastasis

Parameters	Crude OR (95% CI)	AIC	*P*
PDC grade		61.626	< 0.001
G 1	Reference		
G 2	0.038 (0.004, 0.331)		0.003
G 3	0.002 (< 0.001, 0.018)		< 0.001
Depth of submucosal invasion		101.793	< 0.001
> 1 mm	Reference		
≤ 1 mm	15.326 (4.282, 54.855)		
Stratification of submucosal invasion		112.951	0.002
SM 1	Reference		
SM 2	10.303 (2.315, 45.848)		
Tumor size (mm)		127.937	0.566
≤ 20	Reference		
> 20	0.732 (0.252, 2.123)		
TB grade		87.953	< 0.001
Bd 1	Reference		
Bd 2	0.088 (0.022, 0.351)		0.001
Bd 3	0.013 (0.003, 0.063)		< 0.001
Tumor grade (WHO)		125.464	0.319
1	Reference		
2	0.435 (0.095, 1.990)		0.283
3	1.467 (0.125, 17.213)		0.761
PNI		115.270	< 0.001
Absence	Reference		
Presence	0.109 (0.035, 0.339)		
LVI		118.567	0.001
Absence	Reference		
Presence	0.136 (0.042, 0.439)		
Mucinous composition		127.929	0.539
Absence	Reference		
Presence	0.539 (0.660, 0.175)		
Tumor location		124.534	0.943
Right colon	Reference		
Left colon	< 0.001 (< 0.001, –*)		0.999
Rectum	< 0.001 (< 0.001, –*)		0.999
Number of lesions		127.919	0.519
Unifocal	Reference		
Multifocal	0.478 (0.051, 4.500)		
Operate method			0.541
Laparotomy	Reference		
Laparoscopy	0.745 (0.289, 1.918)		

*OR* odds ratio, *CI* confidence interval, *AIC* Akaike information criterion, *PDC* poorly differentiated cluster, *TB* tumor budding, *PNI* perineural invasion, *LVI* lymphvascular invasion

* represent the missing values

**Table 4:** Multivariate analyses of the potential predictors of lymph node metastasis

Parameters	Adjusted OR (95%CI)	*P*
PDC grade		0.004
G 1	Reference	
G 2	0.026 (< 0.001, 2.313)	0.111
G 3	0.001 (< 0.001, 0.139)	0.007
Depth of submucosal invasion		0.034
> 1 mm	Reference	
≤ 1 mm	23.205 (1.266, 425.177)	
Stratification of submucosal invasion		0.095
SM 1	Reference	
SM 2	71.683 (0.476, 10,786.720)	
TB grade		0.106
Bd 1	Reference	
Bd 2	1.509 (0.139, 16.376)	0.735
Bd 3	0.040 (0.001, 1.082)	0.056
PNI		0.039
Absence	Reference	
Presence	0.078 (0.007, 0.882)	
LVI		0.017
Absence	Reference	
Presence	0.061 (0.006, 0.609)	

*OR* odds ratio, *CI* confidence interval, *PDC* poorly differentiated cluster, *TB* tumor budding, *PNI* perineural invasion, *LVI* lymphvascular invasion

Regarding the other LNM predictors, univariate logistic regressions showed that the depth of submucosal invasion (crude OR = 15.326; 95% CI = 4.282, 54.855; *P* < 0.001), stratification of submucosal invasion (crude OR = 10.303; 95% CI = 2.315, 45.848; *P* = 0.002), TB grade (*P*_Total_ < 0.001; crude OR_Bd 2_ = 0.088; 95% CI_Bd 2_ = 0.022, 0.351; *P*_Bd 2_ = 0.001; crude OR_Bd 3_ = 0.013; 95% CI_Bd 3_ = 0.003, 0.063; *P*_Bd 3_ < 0.001), PNI (crude OR = 0.109; 95% CI = 0.035, 0.339; *P* < 0.001), and LVI (crude OR = 0.136; 95% CI = 0.042, 0.439; *P* = 0.001) all influenced LN status (Table 3). Multivariate analysis found that the depth of submucosal invasion (adjusted OR = 71.683; 95% CI = 0.476, 10,786.720; *P* = 0.034), PNI (adjusted OR = 0.078; 95% CI = 0.007, 0.882; *P* = 0.039), and LVI (adjusted OR = 0.061; 95% CI = 0.006, 0.609; *P* = 0.017) independently influenced LNM. See Table 4 for the details.

### Fit comparisons

PDC grade was the best predictor of LNM. The AIC value of PDC grade was the lowest (AIC = 61.626) (see details in Table 3). It was also included in the predictive model. Other variables analyzed were the depth of submucosal invasion, stratification of submucosal invasion, TB grade, PNI, and LVI (model AIC = 49.258) (see details in Table 5).

**Table 5:** Results of backward regression

	Variables	Log likelihood of models	Changes of -2 log likelihood	*P*
Step 1	PDC grade	− 29.753	22.248	< 0.001
	TB grade	− 21.472	5.686	0.058
	Depth of submucosal invasion	− 21.659	6.06	0.014
	Stratification of submucosal invasion	− 20.455	3.651	0.056
	PNI	− 20.938	4.618	0.032
	LVI	− 21.766	6.273	0.012

*PDC* poorly differentiated cluster, *TB* tumor budding, *PNI* perineural invasion, *LVI* lymphvascular invasion

### Prognostic implications for PDC grade

The median follow-up time was 49 months. Patients lost to follow-up patients were faithfully recorded, accounting for 1.1%. Taking OS as the endpoint, the mean follow-up times of the surviving patients were 55 months in the G1 group, 42 months in the G2 group, and 41 months in the G3 group. Using DFS as the endpoint, the mean follow-up times of the surviving patients were 55 months in the G1 group, 45 months in the G2 group, and 40 months in the G3 group. Among the patients with postoperative distant metastasis, 55.6% had liver metastasis, 33.3% had lung metastasis, and 11.1% had bone metastasis. All metastases occurred within 5 years after surgery.

**Fig. 1:**
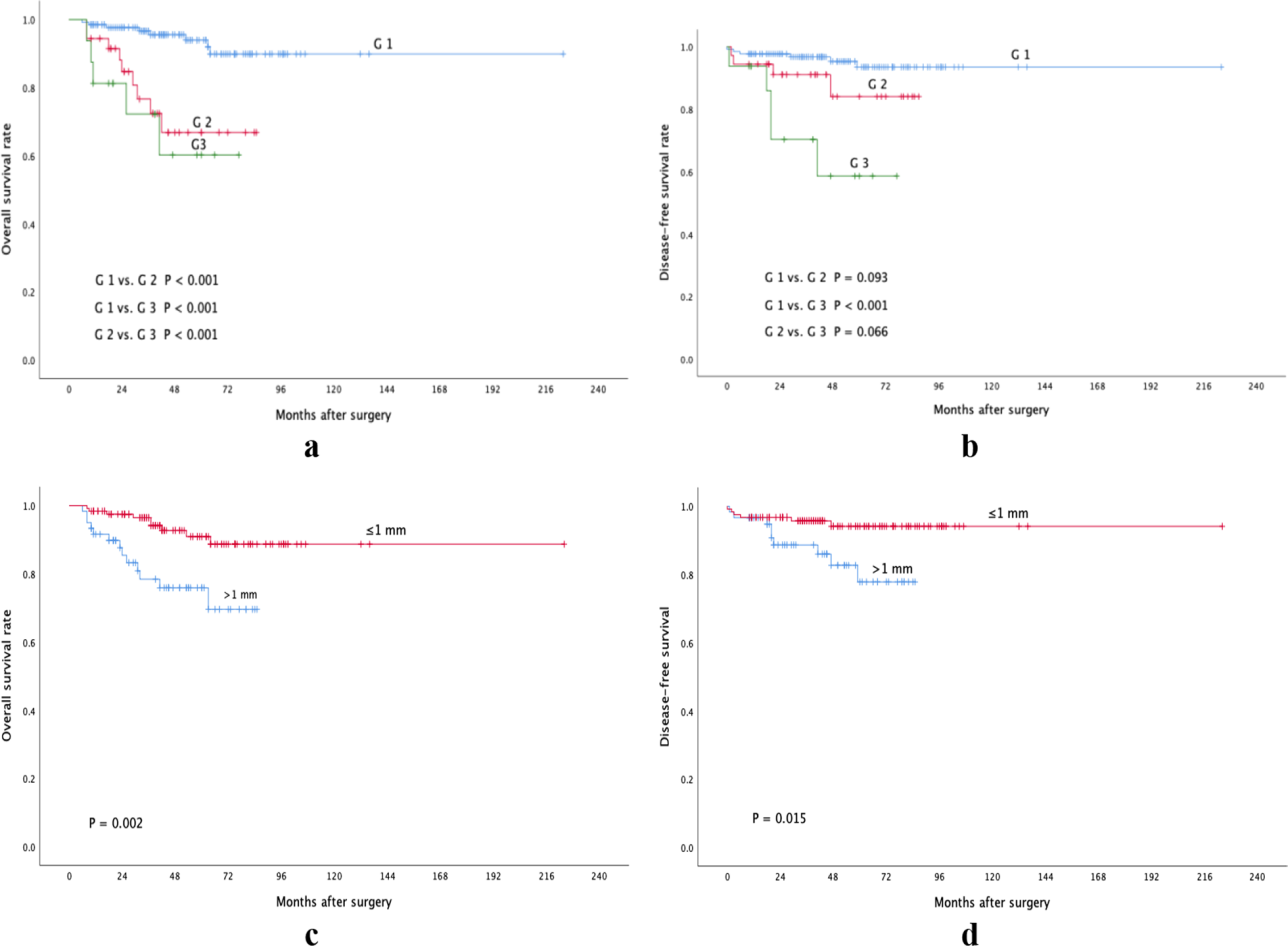
Kaplan–Meier survival curves of patients with T1 colorectal cancer in months (*P* values were calculated by log-rank test). **a** Overall survival curves of poorly differentiated cluster grade; **b** Disease-free survival curves of poorly differentiated cluster grade; **c** Overall survival curves of depth of submucosal invasion; **d** Disease-free survival curves of depth of submucosal invasion

PDC grade was an independent risk factor for negative outcomes. In the univariate analysis, taking OS as the endpoint, 5 patients in the G1 group, 9 in the G2 group, and 5 in the G3 group had died at the 5-year follow-up, and the mean OS times (Table 6) and OS rates (Fig. 1a) of the three groups differed significantly according to the log-rank test. Taking DFS as the endpoint, 6 patients in the G1 group, 4 in the G2 group, and 5 in the G3 group had tumor recurrence/distant metastasis at the 5-year follow-up, and the mean DFS times (Table 6) and DFS rates (Fig. 1b) of the G1 and G3 groups differed significantly according to the log-rank test. The independent predictive value of PDC grade for OS and DFS was determined via multivariate analyses (see Table 7 for the details).

**Table 6:** Survival time of T1 colorectal cancer patients with different poorly differentiated cluster grades

PDC grade	OS (mean ± SD)	DFS (mean ± SD)
G 1	205.773 ± 6.427	211.528 ± 5.103
G 2	65.701 ± 5.401	77.277 ± 4.553
G 3	55.438 ± 7.782	54.516 ± 7.965

*PDC* poorly differentiated cluster, *OS* overall survival, *SD* standard deviation, *DFS* disease-free survival

**Table 7:** Multivariate survival analyses of T1 colorectal cancer patients

Parameters	OS		DFS	
	HR (95% CI)	*P*	HR (95% CI)	*P*
PDC grade		0.015		0.048
G 1	Reference		Reference	
G 2	4.315 (1.506, 12.568)	0.007	2.389 (0.605, 9.439)	0.214
G 3	5.049 (1.326, 19.222)	0.018	6.621 (1.472, 29.786)	0.014
Depth of submucosal invasion		0.353		0.543
> 1 mm	Reference		Reference	
≤ 1 mm	0.614 (0.220, 1.718)		0.666 (0.180, 2.468)	

*OS* overall survival, *DFS* disease-free survival, *HR* hazard ratio, *CI* confidence interval, *PDC* poorly differentiated cluster

Regarding the depth of submucosal invasion, according to the univariate analyses, 12 patients in the > 1 mm group and 8 patients in the ≤ 1 mm group had died at the 5-year follow-up, and the mean OS times (Table 8) and OS rates (Fig. 1c) of the two groups differed significantly according to the log-rank test. Regarding DFS, 9 patients in the > 1 mm group and 6 patients in the ≤ 1 mm group had recurrence or distant metastasis at the 5-year follow-up, and the mean DFS times (Table 8) and DFS rates (Fig. 1d) of the two groups differed significantly according to the log-rank test. Regarding postoperative adjuvant chemotherapy, there were no statistically significant prognoses implemented in the univariate survival analyses (*P*_OS_ = 0.069, *P*_DFS_ = 0.55), and it was not introduced into multivariate Cox regression models. The results of multivariate analyses are shown in Table 7.

**Table 8:** Survival time of T1 colorectal cancer patients with different depths of submucosal invasion

Depth of submucosal invasion	OS (mean ± SD)	DFS (mean ± SD)
> 1 mm	68.318 ± 4.015	73.319 ± 3.532
≤ 1 mm	203.077 ± 6.857	211.974 ± 4.865

*OS* overall survival, *SD* standard deviation, *DFS* disease-free survival

### Interobserver agreement of the PDC grading system

The kappa value between the two pathologists was 0.906, which indicated a strong concordance for PDC grading in our test (see Table 9 for the details).

**Table 9:** Kappa consistency test between our two pathologists for PDC grading

Pathologist B	Pathologist A			Total	Kappa value
	G1 (%)	G2 (%)	G3 (%)		
G 1	124 (98.4)	3 (7.3)	1 (6.3)	128	0.906
G 2	1 (0.8)	36 (87.8)	0 (0.0)	37	
G 3	1 (0.8)	2 (4.9)	15 (93.8)	18	

*PDC* poorly differentiated cluster

## Discussion

The results of this retrospective study comprehensively showed the high clinical value of PDC grade when selecting resection strategies for T1 CRC patients through multiple rounds of validation. PDC grade is an independent predictor for LNM and has the best predictive value. It was also included in the predictive model, providing a foundation for the use of PDC grade as a reference for surgery, simplifying risk stratification models, and refining prior treatment strategies. PDC grade could also be regarded as a predictor of oncological outcomes, and it was found to be an independent risk factor for tumor recurrence, distant metastasis, and patient death. We also considered its correlation with other indexes and high interobserver agreement. In view of its high clinical value and availability, we recommend highlighting its applicability in the clinic and adding it to routine histopathological reports, especially for CRC patients. This work also broadened the methodological approach for similar research; for the chosen index, the fitness of the variables (AIC value) was explored, and backward regression was properly used. The results of this work also indicate that multidisciplinary team (MDT) discussion should be recommended.

Our results revealed a correlation between a higher PDC grade and deeper submucosal invasion. Combined with the findings reported in previous studies, ECRCs with deeper submucosal invasion tend to be incompletely resected under endoscopy and even require adjuvant therapy after rection [34]. Our assumption that CRCs with a high PDC grade need to be surgically resected was indirectly confirmed. An association between PDC grade and TB grade was also observed. Previous studies all showed evidence of epithelial-mesenchymal transition (EMT) [35], which was even speculated to represent different stages of tumor growth [36]. However, their fundamental differences and similarities require further studied. The definitions of the number of cells are worth reconsidering, perhaps even trying to combine them to simplify the risk stratification model. We also found that PDC grade was related to PNI, LVI, and mucinous composition. PNI, LVI and mucinous composition might promote the formation of PDCs, which provides a treatment opportunity. Moreover, Barresi V et, al. pointed out that PDCs might be relate to the biomolecular profiles of CRCs, and gene mutations, such as KRAS mutations, were more common in CRCs with high PDC grades [37]. Therefore, the PDC grade might serve as a reference for clinicians to administer targeted therapies that match the correlation between a high PDC grade and the presence of postoperative adjuvant chemotherapy; however, the mechanisms need to be further clarified in all CRC patients.

The depth of submucosal invasion is the basis of T1 CRC treatments in the Japanese Society for Cancer of the Colon and Rectum (JSCCR) guidelines [38, 39]. Nevertheless, because of the additional time needed to measure depth and the absence/ambiguity of the muscularis mucosae in endoscopic specimens, this indicator has not been fully implemented in Europe. However, the stratification of submucosal invasion is more difficult than the stratification of the depth of submucosal invasion. There are also difficulties in promoting TBs; specific staining can be useful to help distinguish TBs, but excess staining might cause confusion with other tissue cells. The TB grade showed predictive paradoxes in our study and previous studies [20]. We found that TBs were associated with LNM in the univariate analysis (crude OR_Bd 2_ = 0.088, crude OR_Bd 3_ = 0.013); however, an association was not found in the multivariate analysis. Ueno et al. [20] previously reported a similar phenomenon in their study on stage II-III CRC patients. We therefore believe that the difficulty in assessing TBs is the main reason, although the bias caused by our small sample size cannot be ignored. Identification of PNI and LVI in H&E-stained specimens is also difficult [40]. As a considerable prognostic predictor of CRCs [41, 42], high interobserver variability is the main limitation of tumor grade [29, 43, 44]. Postoperative adjuvant chemotherapy, which found a statistically significant difference between patients with/without LNM, was not selected as the risk factor for patients’ LNM due to patients with stage III, and high-risk stage II CRC (with synchronous LNM) were the adaptation sign of postoperative adjuvant chemotherapy [31]. The LN resections were also curable resection in our work, moreover, our mean LNs per patients higher than that recommended by AJCC and National Quality Forum. Therefore, we did not take how the LN resection was performed as a risk factor into our analyses.

The PDC grade performed well in terms of predictive power. PDCs are cell clusters/nests that are larger and recognizable without specific staining [30], which saves time in identification, increases the accuracy of the report, and improves the reproducibility of related laboratory studies. Furthermore, high interobserver agreement was found for PDCs [20, 27–29], making them easier to use in the clinic and reducing training costs. The strong concordance for PDC grading was also found in our work, according to the range of kappa values, validating the effectiveness of PDC as a predictor again. In addition, PDCs was found to be an independent predictive index for LNM after excluding the confounder of the depth of submucosal invasion, which suggests that innovative techniques and devices for endoscopic full-thickness resection may be a promising alternative to major surgery when the integrity of endoscopic resection is questionable and the risk of LNM is low [45, 46]. A high PDC grade has also been previously found to be related to occult LNM [28]. Moreover, PDC grade fit the LNM best both alone and in the model. Therefore, selecting treatment methods mainly depending on the PDC grade might be feasible. Furthermore, although PDC is a pathological indicator, when referencing it to guide diagnosis and treatment strategies for T1 CRC patients, a multidisciplinary team (MDT) discussion, including gastrointestinal surgeons, oncologists, radiologists, and so on, is recommended to make comprehensive decisions. Patient age, tumor location (colon or rectum), and common comorbidities all need to be seriously considered.

Obviously, to obtain firm conclusion that PDC grade is a good reference for selecting management strategies, investigating its predictive value relative to LNM is not enough. Some research has found that local LNM is a precursor for distant metastases [15] and that 0.3–4.5% of patients develop metastases after LN dissection [14, 47]. Performing LN dissection did not improve the oncological outcomes in these cases. Therefore, we further investigated the prognostic value of the PDC grade in detail. Our results showed that PDC grade is an independent risk factor for a poor prognosis in T1 CRC patients; moreover, patients with different PDC grades had different prognoses. Moreover, although the presence of postoperative adjuvant chemotherapy was related to a high PDC grade, it was not analyzed as a covariate in our multivariate survival analyses because it was not found to be a predictor of overall and distant-metastasis survival in our univariate analyses. Considering the mean survival time (OS, 206 months; DFS, 212 months), the average age when initially diagnosed (70 years old), and the cutoff time commonly used for oncological outcomes in the clinic (5 years), we initially speculated that G1 CRC patients do not require adjuvant therapy after tumor resections. For patients with G2 CRC, the mean survival time dropped sharply at approximately 5 years; thus, clinicians must be vigilant and follow them closely, providing adjuvant radiochemotherapy when necessary. G3 CRC patients had the shortest survival time; consequently, they needed the most extensive treatment and the closest postoperative monitoring, even though some studies found no significant relationship between monitoring intensity and postoperative recurrence [48]. Further research is needed to clarify the proper posttreatment monitoring frequency.

The procedure used to explore predictive indicators in our work is well established and provides a reference for developing new indexes. We applied the AIC value to compare fit and prove its clinical utility, expanding the use of the fit comparative method of indicators and models. Backward regression was also properly and innovatively used. Moreover, our concordance for the indicator grading was also found by the kappa consistency test. We await the exploration and validation of image recognition tools based on deep learning to further avoid biases caused by histological evaluation.

This work has unique clinical value. However, we acknowledge that, as a single-center retrospective study, the limitations of the study type cannot be ignored. Moreover, the small sample size and the low absolute number of patients with oncological outcomes (LNM and oncological endpoints) could have caused bias. Nevertheless, we faithfully recorded the patients’ information in detail. Moreover, all slides were reevaluated according to the definitions in the available consensus to improve the quality of the pathological records. In addition, we used archived slides, and tissue processing was not standardized. However, this defect exactly reflects real-time practice and increases the generalizability of our results to a certain extent. Furthermore, our participants were all from a registered population in Qingdao and the surrounding area; therefore, extrapolating our results requires caution. The interstitium of participants might promote standardized sample collection and minimize possible residual confounding caused by differences in subjects’ genetic backgrounds. Moreover, this study included only surgical resection cases when the endoscopic resection alone cohort was essentially part of our true target. However, potential biases such as insufficient material and blurred margins caused by initial endoscopic resection and the possibilities of unclarified depth/stratification of submucosal invasion and PNI/LVI caused by unradical endoscopic resection followed by secondary surgical resection were avoided to some extent. Further investigations based on endoscopic resection cases alone are still needed.

To further confirm our conclusions, multicenter, prospective, and large sample studies are needed. Moreover, the predictive value of PDC grade in biopsy samples also requires further investigation. This might represent a clinical window that allows clinicians to stratify patients by their LNM risk and prognosis at initial biopsy. Then, the subsequent management of patients may be improved. Furthermore, more complete stratification models based on methods such as machine learning are also expected in the future.

## Conclusions

PDC grade is the best predictive factor for LNM and the oncological outcomes of T1 CRC patients, representing a novel, simple and reliable surgical indicator.

## Data Availability

The datasets generated during and/or analysed during the current study are available from the corresponding author on reasonable request.
